# Correlations of Tinel and Phalen Signs with Nerve Conduction Study Test Results in a Randomly Chosen Population of Patients with Carpal Tunnel Syndrome

**DOI:** 10.3390/neurosci6040094

**Published:** 2025-09-28

**Authors:** Katarzyna Kaczmarek, Jędrzej Pepliński, Anna Kaczmarek, Dariusz Andrzejuk, Kacper Andruszkiewicz, Alicja Wysocka, Matylda Witkowska, Juliusz Huber

**Affiliations:** 1 Department of Pathophysiology of Locomotor Organs, Poznań University of Medical Sciences, 28 Czerwca 1956 No 135/147, 61-545 Poznań, Poland; katarzynakaczmarek@ump.edu.pl (K.K.); peplinski.jedrzej@gmail.com (J.P.); kacper.andruszkiewicz@wp.pl (K.A.); alicja.wysocka00@gmail.com (A.W.); matylda.wit@gmail.com (M.W.); 2 Faculty of Medicine, Warsaw University of Medical Sciences, Żwirki i Wigury 101, 02-089 Warsaw, Poland; akr.kaczmarek@gmail.com (A.K.); andrzejukbusiness@gmail.com (D.A.)

**Keywords:** carpal tunnel syndrome, median nerve, Phalen’s test, Tinel’s test, clinical diagnosis, electroneurography, sensory conduction studies, motor conduction studies

## Abstract

Background: The consequences of median nerve compression at the carpal tunnel level require a precise diagnostic evaluation before a frequently applied surgical intervention. Positive Tinel or Phalen signs are not always related to abnormal results in electroneurographic examinations of sensory and motor nerve fibers, which are intended to confirm final diagnoses, thereby confusing both surgeons and neurophysiologists. In the face of contradictory data, this study aims to reinvestigate these correlations in a randomly chosen population of patients with a primary diagnosis of carpal tunnel syndrome (CTS). Methods: Seventy-five randomly chosen patients with clinically detected CTS underwent neurophysiological studies of median nerve sensory (SNAP) and motor (CMAP) fibers conduction at the wrist. Both the median and ulnar nerves were assessed to reduce the risk of misinterpretation related to anatomical variations. Results: This study provides evidence on the relatively high utility of Phalen’s test in the early clinical detection of CTS within a general population of patients, whose positive results moderately correlate (rho = −0.327) with abnormalities in amplitudes rather than the distal latency parameters of SNAP recordings. The axonal injury type is more distinct than slowing-down impulses at the wrist following compression of the sensory nerve fibers in the early course of CTS. Positive Tinel’s test results are useful in diagnosing CTS patients with advanced axonal and demyelinating changes in the motor fibers at the wrist, which weakly correlate with prolonged latency and decreased amplitude in SNAP recordings (rho = −0.214 and rho = −0.235, respectively), but not with abnormalities in recordings of both amplitudes and latencies in CMAP electroneurography. Conclusions: The correlations between clinical signs and neurophysiological findings in CTS indicate that provocative tests, such as Phalen’s and Tinel’s, have limited diagnostic value, demonstrating only weak-to-moderate associations with neural conduction parameters. A positive Tinel’s sign should be regarded mainly as a marker of severe or chronic sensory impairment, often accompanied by motor fibers involvement in advanced pathological stages, rather than as an indicator of motor damage alone. Nerve conduction studies remain essential for confirming CTS, assessing its severity, and guiding treatment decisions, including surgical qualification. The presented correlation of clinical and functional neurophysiological results in CTS diagnosis allows us not only to specify the source and severity of the pathology of the median nerve fibers but also may influence the personalization of physiotherapeutic and surgical treatments.

## 1. Introduction

Carpal tunnel syndrome (CTS) is a mononeuropathy associated with the compression of both the sensory and motor median nerve fibers in the wrist and pre-wrist areas [1,2]. Most cases of CTS are not associated with anatomical anomalies within the canal [3] or systemic disease [4]. A physiological condition such as pregnancy can predispose patients to CTS due to hormonal changes [5]; other risk factors are working with a computer or sports [6,7,8,9,10]. However, some medical conditions and comorbidities can promote or directly cause CTS symptoms, like rheumatoid arthritis, diabetes mellitus, hypothyroidism, obesity, alcoholism, chronic renal failure, collagenosis, acromegaly, Down syndrome, and amyloidosis [11,12,13]. CTS may co-exist with other pathologies in the upper extremity, such as De Quervain’s disease, Raynaud’s syndrome, flexor tendonitis, and ulnar Guyon tunnel syndrome, among others, which can make differential diagnosis difficult [14,15]. A relationship between abnormalities in median and ulnar nerve conduction is often reported; conduction impairment of the ulnar nerve increases with increasing median nerve involvement severity [16]. A relationship between physical examinations and the nerve conduction of the CTS in patients with diabetic polyneuropathy has been reported [17].

The prevalence of CTS in the world population has been described to vary from 3 to 23%, with a predilection more for women than men [18,19,20].

CTS results in neurological symptoms, including pain and sensory disturbances along the distribution of the median nerve in the fingers, as well as weakness and atrophy in the thenar muscles [21]. A set of these characteristic clinical symptoms is generally the key to diagnosis. Numbness and tingling occur owing to the innervation of the median nerve; another common symptom reported by patients is nocturnal paresthesia [8,22].

In the clinical diagnostic process of CTS, the most common evaluations include Tinel’s and Phalen’s provocative tests, which provide symptoms of increased pain and sensory disturbances; however, despite being easy to administer, they often lack definitive diagnostic significance [23,24,25]. The sensitivities of these tests in CTS detection are disputable [22,26,27]. Neurophysiological examination plays a supplementary role in clinical CTS diagnosis, with electroneurographical examination (ENG) being the most commonly used, based on the electrical stimulation of the upper extremity sensory and motor nerve fibers [28]. Notably, a positive Phalen or Tinel test alone cannot be considered a confident diagnosis of CTS. The use of other diagnostic tools, such as electroneurophysiological or neuroimaging diagnostics, is required for a comprehensive evaluation of the disease process. These tests can be combined with magnetic resonance imaging (MRI) to visualize the structures of the wrist and identify any abnormalities, such as swelling or compression of the median nerve within the carpal tunnel [29,30]. Attempts to incorporate machine learning algorithms into evaluations with ENG diagnostics may provide a valuable tool for clinicians in the diagnosis and management of CTS [31]. However, despite many articles of this type, there is still a lack of studies directly comparing how specific neurophysiological parameters, such as latency and amplitude, and specifying how particular ranges of these parameters relate to the results of clinical tests, for example, Tinel’s or Phalen’s tests [32,33,34]. Moreover, detailed studies on this issue do not seem to describe random populations of CTS patients, including those with subclinical symptoms, which are the most frequent cases in neurological and orthopedic practice.

There is a general agreement on the effectiveness of ultrasonography in evaluating CTS consequences, but not its grading [35,36]. The diagnostic accuracy of neurodynamic tests used to detect carpal tunnel syndrome in patients with unilateral symptoms has been examined [37], and it is evident that healthcare professionals still expect simple and precise diagnostic tools for detecting and grading CTS in order to make final decisions about continuing conservative treatment or undertaking surgery [38].

Electrophysiological severity classifications of CTS using nerve conduction studies (NCSs) have also been reported, and there are many contradictory reports on the relationship between severity classifications and clinical symptoms [18,39,40,41], except for cases of patients with advanced axonal-type neuropathy in motor fibers of the median nerve [42].

Severity classification methods based on electrodiagnostic tests have been developed [43,44], among which Steven’s classification method has been widely used [45]. CTS is classified as mild when there is delayed distal sensory latency or a decrease in the amplitude of the sensory nerve action potential (SNAP). It is classified as moderate in cases with delayed distal motor latency in the compound motor action potential (CMAP). Finally, for severe cases, the classification is determined by the absence of a SNAP response, a decrease in CMAP amplitude, or the observation of denervation potentials in needle electromyographic recordings. Padua’s neurophysiological classification includes extreme CTS (absence of median motor and sensory response); severe CTS (absence of SNAP; abnormal CMAP motor latency); and moderate CTS (abnormal SCV results; abnormal CMAP latency value) [44,46]. However, clinical neurophysiological practice often reveals CTS patient cases when motor nerve fiber damage appears long before muscle atrophy, and sensory nerve damage does not necessarily occur first [47]. Vázquez-Sánchez et al. [48] provided a practical algorithm for ENG referral, taking into account clinical symptoms, demographic factors, and parameter variations in ENG tests, basing the results on a large population of symptomatic and asymptomatic CTS patients.

The inconsistency of data regarding CTS severity scales and the divergent conclusions drawn from clinical and neurophysiological comparisons may, at least in part, result from the preliminary selection of patients at specific stages of disease advancement in previous studies.

Therefore, clarifying the diagnostic value of simple clinical tests in relation to objective neurophysiological parameters is crucial. Such knowledge may improve the accuracy of CTS diagnosis in everyday practice and support more rational decisions regarding conservative versus surgical management.

This study aimed to reinvestigate the correlations between clinical test outcomes and neurophysiological findings in a randomly selected cohort of patients with confirmed CTS across different severity grades, representing a novel approach.

## 2. Materials and Methods

### 2.1. Participants, Study Design, and Clinical Evaluation

Data for this research were collected during routinely performed electroneurographic diagnostic nerve conduction studies of patients at the Wiktor Dega Orthopaedic and Rehabilitation Clinical Hospital in Poznań, Poland. The same clinical tests and neurophysiological diagnostics used to detect carpal tunnel syndrome (CTS) were conducted once in 75 patients, and similar studies were performed on a control group of 75 healthy volunteers. Data from ENG parameters from the control group (N = 75) were compared with similar recordings in the CTS group (N = 75). We aimed to identify statistically significant abnormalities in nerve impulse conduction in median nerve fibers in patients whose clinical examination results did not always reveal pathological symptoms. Patients were randomly selected from the hospital database covering the years 2023–2024, using a computer-generated sequence. Control group data were collected during a periodic study at the same facility; this study is performed every 5 years on a population of 100 healthy subjects of both genders with different demographic and anthropometric properties to actualize the reference values of neurophysiological parameters used for diagnostic purposes. Ethical considerations aligned with the Declaration of Helsinki. The approval of the Bioethics Committee of the Poznań University of Medical Sciences was also obtained (including studies on healthy people, decision no. 554/17). All subjects understood that there was no special financial benefit from participation; they signed a written consent form for voluntary attendance in the study and permitted the results to be disseminated, maintaining the anonymity of their personal data. The demographic and anthropometric characteristics of the subjects are presented in Table 1. Subjects belonging to the two groups did not significantly differ.

The main inclusion criteria for patients were clinical and neurophysiological symptoms of CTS. Clinical symptoms with a duration longer than 3 months on average included uni- or bilateral disturbances in the sensory perception of the index finger, detected using the tactile method with Von Frey’s filaments (Semmes–Weinstein monofilaments) [49,50]; uni- or bilateral pain in the same hand area reported on a 10-point visual analog scale (VAS; 0—no pain, 1–3—mild pain, 4–6—moderate pain, 7–10—severe pain) [10,51], mainly of the nocturnal nature; and possible positive Tinel’s and Phalen’s signs detected unilaterally. Neurophysiological symptoms of positive CTS were uni- or bilateral abnormalities in the parameters (amplitudes and/or latencies) of the sensory or motor electroneurographic findings from median nerve examinations, with reference to the normative values established in controls [52]. Positive results on the clinical and electroneurographic findings were contraindications for the asymptomatic healthy volunteers to be included in the control group.

The main exclusion criteria for all subjects were a history of wrist and hand surgery, oncological episodes, inflammatory disease, pregnancy, electronic implants such as pacemakers and cochlear implants, head injury, stroke, epilepsy episodes, mental disorders, cardiovascular disease, myelopathy, degenerative changes in the cervical spine (after verification on CT or MRI scans), other peripheral neuropathies from the level of arm (verified in electroneurographical studies), de Quervain syndrome, trigger finger, and COVID-19-related neurological symptoms [53]. None of the studied patients had any past medical history of comorbidities, including rheumatoid arthritis, diabetes, hypothyroidism, obesity, alcoholism, chronic renal failure, collagenosis, acromegaly, Down syndrome, or amyloidosis.

The study design includes presentation results from non-invasive studies on only the symptomatic upper extremities of the CTS patients (all right-handed) in comparison with the results from the right extremities of the control subjects (69 healthy subjects were right-handed; only 6 subjects were left-handed). When 75 patients were examined, some had bilateral symptoms of CTS, and finally, a total of 112 symptomatic hands were separately analyzed. All clinical studies and provocative tests for CTS detection were performed and evaluated by a single specialist in neurology to ensure maximum objectivity in the physical examination. The same physician completed medical histories regarding the development of this disease; occupational activity risk; uni- or bilateral symptomatology; and attempts at conservative treatment, including the corticosteroid injections in the wrist.

**Figure 1:**
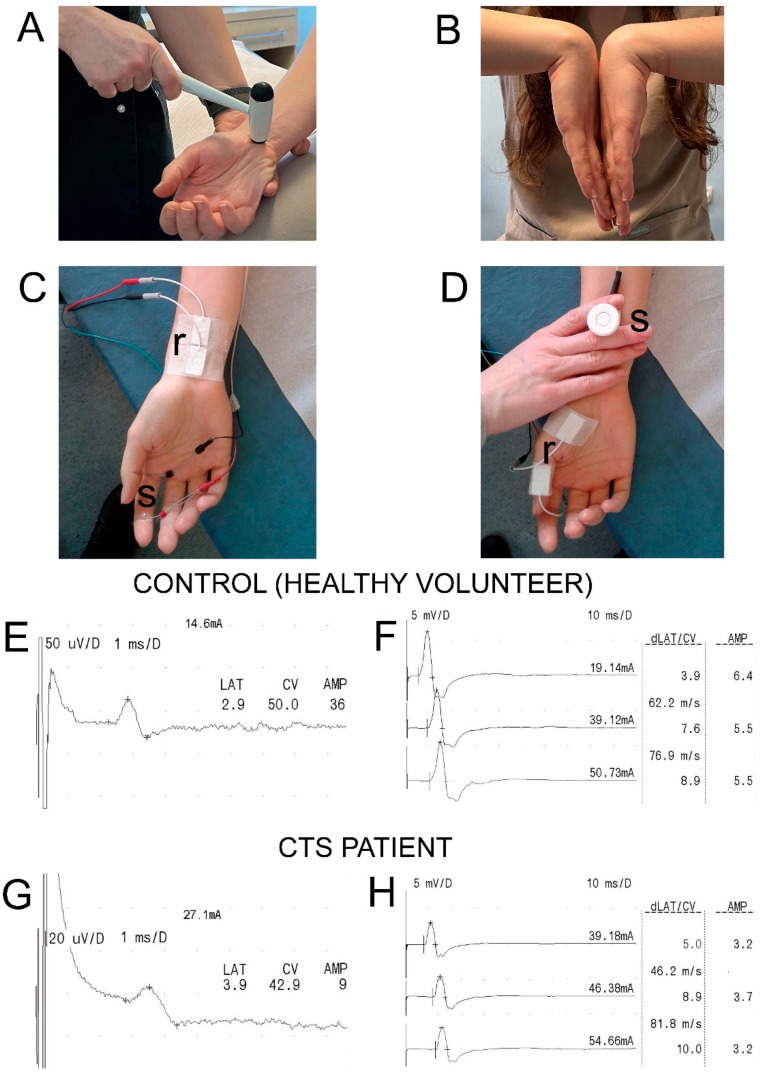
Methodological aspects of Tinel’s (**A**) and Phalen’s (**B**) tests used for the clinical diagnosis of carpal tunnel syndrome (CTS). Sites of recordings (r) with the surface electrodes following stimulation (s) with a single electrical stimulus over the anatomical passage of the median nerve for SNAP in SCV studies and CMAP in MCV studies are presented in (**C**,**D**), respectively. Examples of SNAP recordings in (**E**,**G**) and CMAP recordings in (**F**,**H**) were performed on one of the control subjects (upper traces) and one of the CTS patients (lower traces), respectively. Note twice as less comparing in G and E, and similar comparing F and H amplifications of the recordings. Time bases in E and G, and in F and H are similar. Note that strengths of applied stimuli (in mA) to evoke potentials in CTS patients are larger. Latencies of recordings are scaled in ms, conduction velocities in m/s and amplitudes in micro- (**E**,**G**) or millivolts (**F**,**H**). Abbreviations: CTS—carpal tunnel syndrome, SNAP—sensory nerve action potential, SCV—sensory conduction velocity, CMAP—compound muscle action potential, MCV—motor conduction velocity, LAT or dLAT—distal latency, CV—conduction velocity, AMP—amplitude.

Experienced neurophysiologists performed electroneurographic tests on all subjects. Each patient underwent Tinel’s and Phalen’s provocative tests for the diagnosis of CTS prior to electroneurography recordings [26]. To perform Tinel’s test (Figure 1A), the symptomatic hand was tapped three times on the median nerve near the tendon of the palmar longus muscle on the palmar side of the wrist using a Luxamed 07E neurological hammer (Luxamed, Blaubeuren, Germany). If the tapping evoked a sensation of paresthesia and pain on the anatomical passage of the median nerve, the test result was considered positive [54]. A positive Tinel’s sign was understood to be a consequence of median nerve sensory fibers impairment, as well as severe motor fibers injury associated with visible thenar muscle atrophy and only a weak voluntary muscle contraction. During Phalen’s test (Figure 1B), the patient was asked to flex both wrists to the maximum and hold them in the flexed position for about 60 s. If paresthesia or pain were reported in the median nerve with innervation of the thumb, index finger, middle finger, half of the ring finger, or forearm, the test results were considered positive [23].

### 2.2. Nerve Conduction Studies (NCSs)

Neurophysiological tests were performed with a four-channel Keypoint System (Medtronic A/S, Skovlunde, Denmark) while the subjects were in supine positions. We followed the guidelines of the European Chapter of the International Federation of Clinical Neurophysiology [10,55] and Stevens [45], as well as the criteria defined by the American Association of Neuromuscular and Electrodiagnostic Medicine (AANEM) [56]. An air-conditioned room with an average temperature of 22 °C was used to perform all tests. Standard, disposable Ag/AgCl surface-recording electrodes (5 mm^2^ of an active surface), with an active electrode placed on the muscle’s belly and a reference electrode placed on the distal tendon of the same muscle (abductor pollicis brevis, Figure 1D) were used to record compound muscle potentials (CMAPs).

Potentials were evoked following the electrical stimulation of the median nerve motor fibers at the pre-wrist anatomical passage with a bipolar stimulating electrode. Similar surface electrodes were used to record sensory nerve action potentials (SNAPs, Figure 1C) when they were placed longitudinally on the middle pre-wrist area. Bipolar ring electrodes were applied to the middle finger for the antidromic excitation of the median nerve sensory fibers. A ground electrode was placed on the dorsal aspect of the hand near the recording electrodes, both during CMAP and SNAP nerve conduction studies.

Electroneurography (ENG) included the assessment of peripheral neural transmission in the motor and sensory fibers of the median nerves bilaterally following the application of single electrical, rectangular pulses with a 0.2 ms duration at 1 Hz, with a delivered intensity of 5 to 80 mA. Recordings were made at an amplification of 5–10,000 µV and a time base of 1–10 ms. Due to the small amplitude parameter, SNAPs required averaging following the application of 20 electrical stimulations. In general, ENG studies used 20 Hz low-pass and 10 kHz high-pass filters during recordings.

The same stimulation principles (medially at the wrist level or at the fifth fingertip) and recording principles (from the abductor digiti minimi or medially from the pre-carpal region) during CMAP and SNAP recordings were used for the ENG ulnar nerve conduction studies.

### 2.3. Statistical Analysis

Statistica version 13.1 (StatSoft, Kraków, Poland) was used to analyze the collected data. Measurable variables were presented with the descriptive statistics as minimal and maximal values (range), the mean or median, and standard deviations (SDs). The normality of distributions was evaluated using the Shapiro–Wilk test, and the homogeneity of variances was characterized using Levene’s test. As most variables did not meet the normal distribution, non-parametric methods were applied. Results recorded in healthy volunteers (controls, N = 75) and patients with carpal tunnel syndrome (CTS, N = 75) were compared based on descriptive statistics. Data comparison was performed with the Mann–Whitney U test, and *p*-values < 0.05 were considered statistically significant.

To determine the required sample size, we used Statistica 13.1 (StatSoft, Poland), employing the Sample Size and Power Analysis module. The calculation was based on the primary outcome variables of the SNAP and CMAP amplitudes and latencies recorded and stimulated at the pre-wrist stimulation site from the first twenty subjects in the pilot study. Assuming a moderate correlation of r = 0.30, with a two-tailed α = 0.05 and a statistical power of 0.80, the minimum required sample size was estimated at N ≈ 85 hands. A total of 112 symptomatic hands were analyzed, exceeding this threshold and ensuring adequate statistical power. At least 60 subjects were needed for this study. Data mining was conducted to match patients and healthy volunteers based on age, sex, and basic anthropomorphic properties like weight and height. The rationale for performing the comparative studies on an equal number of CTS patients (N = 75) and controls (healthy subjects; N = 75) was the high, compatible number of participants for the purpose of reliable statistical analysis. Finally, we measured equal symptomatic upper extremities (n = 112) from the CTS patients and non-injured upper extremities (n = 112) from the controls.

We used the non-parametric Spearman’s rank correlation coefficient (rho) to examine relationships between clinical provocative tests and nerve conduction study results. A significance level of *p* < 0.05 was chosen to indicate a statistically significant rank correlation. The significant association between the two clinical test results was confirmed with Fisher’s exact test.

## 3. Results

Among the clinically examined 75 patients with diagnosed CTS, 98% had moderate abnormalities in the sensory perception of the index finger and experienced an average pain of 4 on the VAS scale, at least unilaterally. We detected positive unilateral or bilateral Phalen’s and/or Tinel’s signs in the majority of patients.

The 75 patients with detected abnormalities in clinical tests differed from the 75 healthy volunteers in the electroneurographic recording parameters (Table 2).

Significant functional abnormalities in nerve impulse conduction in median nerve fibers during electroneurographical recordings were more common in sensory fibers (41%) than in motor fibers (4%) due to the inability to record more SNAP than CMAP, respectively. An advanced axonal injury in the sensory fibers of the median nerve was also evidenced by the fact that, in the currently analyzed recordings for CTS patients, the SNAP amplitude parameter showed the greatest differences (*p* = 0.009) compared with the controls. The other differences in CMAP recordings parameters and SNAP latencies were *p* = 0.04–0.03.

**Figure 2:**
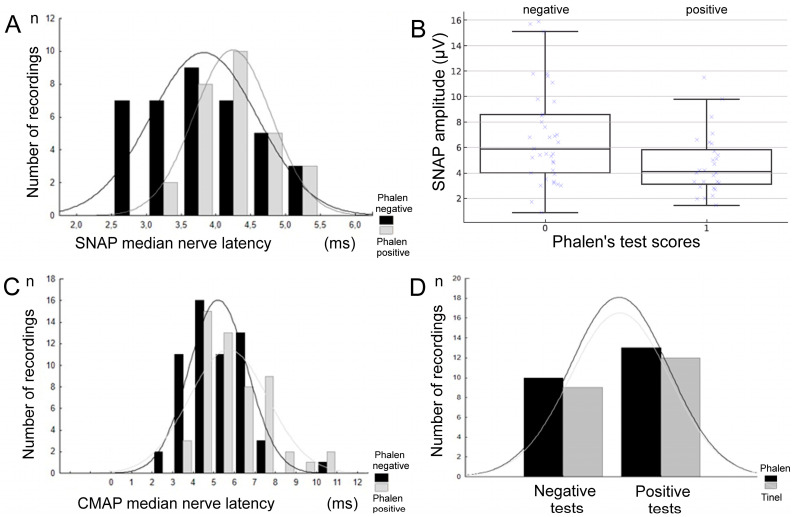
(**A**). Graphical presentation of the relationships between the distribution of SNAP latency parameters and the incidence of the negative and positive Phalen’s test results. (**B**). Distribution of values of SNAP amplitudes stratified by positive and negative Phalen’s test results. (**C**). Distribution of CMAP latency parameters and the incidence of negative and positive Phalen’s test results. (**D**). Coincidence of Phalen and Tinel test results for CTS detection over 112 trials. Abbreviations: SNAP—sensory nerve action potential; CMAP—compound muscle action potential.

Among all performed SCV tests (n = 112), only 66 SNAPs were recorded. Among patients whose SNAP recordings confirmed CTS, 47% had a positive Phalen’s test result, and 35% had a positive Tinel’s test result (Table 3). In patients with a frequent positive and negative Phalen’s test, SNAP latency was most often recorded in a range of 4–5 ms (see Figure 2A). Negative Tinel’s test results were detected the most frequently when SNAP latencies ranged from 4 to 5 ms as well. A distributional shift in SNAP latencies by approximately 1 ms toward lower values, comparable to physiological conduction, was observed in patients with negative Phalen’s test results (*p* = 0.002). A positive Phalen’s test was also most frequently detected when the latencies of recorded CMAPs were in a range of 4.5–7.0 ms (Figure 2C).

Among 112 samples of recordings performed for CTS detection, co-occurrence of positive Tinel and Phalen tests was frequently detected in 27 cases (*p* = 0.002; Figure 2D and Table 4). Co-occurrence of both negative results was noted in 45 patients, and statistical analysis using Fisher’s exact test confirmed a significant association between the two tests (*p* = 0.0027). The calculated odds ratio (OR = 3.62) indicated that patients with a positive Phalen’s test had approximately 3.6 times higher odds of obtaining a positive Tinel’s test result compared with those with a negative Phalen’s test.

Among the 75 CTS patients, a weak but statistically significant negative correlation was found between positive Phalen’s test results and increased CMAP distal latency (rho = 0.197; *p* = 0.03). In SNAP recordings, positive Phalen’s test results were moderately correlated with a decrease in amplitude values (rho = −0.327, *p* = 0.0008) (Figure 2B).

Positive Tinel’s test results were moderately correlated with prolonged latency and decreased amplitude parameters in SNAP recordings (rho = −0.214, *p* = 0.02; rho = −0.288, p = 0.002, respectively). Correlation studies did not yield statistically significant results between positive Tinel’s test scores and either amplitude or latency abnormal parameters in the CMAP recordings.

## 4. Discussion

The most important findings from this reinvestigation of clinical and neurophysiological comparative tests in randomly chosen patients from the general CTS population are abnormalities in nerve impulse conduction in median nerve fibers, more in SNAP (41%) than in CMAP (4%) recordings. An advanced axonal injury in sensory fibers can be seen from the SNAP amplitude parameter, which showed the greatest differences (*p* = 0.009) compared with the controls. These abnormalities may indicate structural changes in sensory fibers rather than motor fibers in the general population of CTS patients, as their smaller diameter fraction is more sensitive to the effects of compression at the carpal tunnel level, consistently with anatomical studies [57,58,59,60]. These studies found that because sensory fibers constitute a large proportion of large myelinated fibers, they are more susceptible to ischemic damage. Moreover, among patients whose SNAP recordings confirmed CTS, only 47% had a positive Phalen’s test, and only 35% had a positive Tinel’s test (see Figure 2D). These data undoubtedly highlight the low sensitivity of Phalen’s test and the even lower sensitivity of Tinel’s test in clinically detecting CTS, confirming their limited diagnostic reliability compared with ENG results.

Seror [61] came to contradictory conclusions compared with our observations about a statistically significant relationship between the severity of electrophysiological changes and the probability of a positive Phalen’s test, which was negative in 34% of CTS patients in his study. Surprisingly, he also found a positive Tinel’s sign in 63% of patients with electrophysiologically proven advanced CTS symptoms, which was also positive in 45% of his control patients [25]. Glass and Ring [62], similar to our findings, concluded that sensory velocity tended to decrease in patients with pathological Phalen’s scores. A review of various reports on this issue [44,45,46,47] did not reveal strong correlations between the results of clinical and neurophysiological examinations in patients with CTS whose disease involved more sensory than motor fibers and whose Phalen’s test results were positive. Our correlation study results for Phalen’s test with SNAP parameters revealed a weak relationship (rho = −0.327), which may be insufficient to be considered clinically meaningful in a random population of CTS patients; however, it shows the most common reality in clinical practice.

Few studies evaluating the clinical effectiveness of the Phalen and Tinel tests address their specificity; usually, their positive scores were considered criteria for including patients with CTS. Our results show that the results of Phalen’s test do not correspond to advanced axonal-demyelinating changes in the sensory fibers of the median nerve, or the positive Tinel’s sign, which is recognized only in cases of advanced motor fiber injury.

Mondelli et al. [20] performed a similar study, in which they split the parameters of motor latency and conduction velocity of sensory fibers in the median nerve into five groups, with a score of 1 indicating a mild pathological state and 5 the most severe. They analyzed a larger group of patients and determined that diagnosing CTS through provocative testing had the highest incidence, with a motor latency value above 4.5 ms (see Figure 2C). The more severe stages of CTS they considered, the lower the chances of provocative testing meeting a proper diagnosis. Sasaki et al. [47] criticized the existing CTS electrophysiological scales, showing that they are constructed in such a way that it is impossible to classify some CTS patients. Saggar et al. [63] proved that mild CTS can be diagnosed as a false negative. However, their results contradict our observations and the findings of other researchers [17,20,62,64], in that for patients without recordable SNAP or severely reduced CMAP, the provocative test achieves the best diagnostic potential. When neurophysiological variables are affected, Tinel’s sign is positive in 59% of cases, while Phalen’s sign is positive in 37.2% of cases. The likely causes of discrepancies in these reports include varying degrees of CTS severity, overlapping neuropathies in long nerves other than the median, and abnormalities in nerve impulse conduction in the C5-C7 spinal root fibers, all of which are not present in the anamnesis, subclinically, or have not been fully diagnosed.

Our study showed the low reliability of the Tinel test in diagnosing CTS compared with recorded SNAP latency, in contrast to the positive results associated with an increase in SNAP latency values for positive Phalen test scores. Presumably, if more patients were tested, similar results would be observed, showing an increase in the number of positive outcomes as latency exceeds 4 ms (Figure 2C). Many scientific papers report very different results for clinical tests, although these studies have often been conducted on groups of a similar size [44,45,46,47]. Notably, even pooled studies in meta-analyses can show significant differences, which presumes the clinical relevance of provocative tests. Provocative tests have been deemed more effective in detecting CTS in moderate-to-severe cases. Consequently, abnormal neurophysiological variables identified through NCS play a crucial role in grading mild CTS cases, which may be mistakenly perceived as normal results in a clinical setting. According to the classification created by Vázquez-Sánchez et al. [48], patients with grade ≥ 3 CTS demonstrate a relationship between typical CTS symptomatology, age over 50 years, male gender, and Phalen’s maneuver and Tinel’s sign positivity, as well as abnormalities in median nerve impulse transmission. Our study partially confirms these observations, mainly in women with CTS of a similar age (57 years on average), providing additional correlation coefficients for clinical and neurophysiological tests.

Previous neurophysiological research data in CTS detection have always been supplemented by data from needle electromyography, which facilitates the qualitative and quantitative assessment of motor unit dysfunction in the abductor pollicis brevis muscle and the degree of denervation resulting from damage to the motor fibers of the median nerve. In the current study, elementary (needle) electromyography was not used, which is a limitation. However, it should be remembered that from the very beginning, the study design was based on a non-invasive approach to assessing patients with CTS. Abnormal electromyographic and clinical signs in CTS are sometimes explained by anatomical variability in the median nerve, its motor branch, and its palmar cutaneous sensory branch, as well as its connections to the ulnar nerve (Martin–Gruber anastomosis) [65]. This very rare connection can complicate the interpretation of nerve conduction studies, leading to misdiagnosis or underestimation of severity. In our study, we examined both the median and ulnar nerves to reduce the risk of misinterpretation related to anatomical variations. Another limitation of our study is the lack of comparison with similar studies performed in other countries. The frequency of CTS, the degree and pace of its development, and even the predominance of its axonal form (in motor fibers, resulting in muscle atrophy) over the axonal-demyelinating form (manifested by a distinct reduction in nerve impulse conduction) may depend not only on demographic but also on regional factors. In our study, a control group with a description and comparison of electroneurographic examination characteristics was included to demonstrate whether, and to what extent, advanced pathological changes occurred in a population with CTS (see Table 2). This procedure is routinely used in comparative statistical analyses, where the intention is to highlight the possibility of demographically determined differences in normative values, i.e., the characteristics of electroneurographic data and reference values in different countries may differ. Their presentation indirectly allowed for a comparison of the results of correlational studies by other authors with those described in the present study.

From a clinical perspective, the value of our results lies primarily in describing the functional state of impulse conduction in the median nerve in the general patient population due to the intentional randomization of the study group, excluding frequently co-occurring comorbidities, which represent the most frequently diagnosed subjects. This data is especially desirable for clinical practitioners. Such an approach was undertaken in early descriptions of this issue by Padua et al. [44], Werner and Andary [28], Lew et al. [33], and Sandin et al. [34]. The presented data provide reliable and comparable data that not only allows for gradation of the degree of damage to the median nerve fibers—especially sensory rather than motor ones—but also enables decision-making in doubtful cases of CTS regarding the need to discontinue conservative treatment and opt for surgical decompression.

Targeted symptomatic treatment, depending on the severity of the condition and its resolution, is possible on the basis of combining data from clinical and neurophysiological studies. Axonal changes that dominate CTS, as in our observations, should be treated based on electrotherapy of the sensory and motor fibers in the median nerve, as recommended by Hernández-Secorún et al. [66]. Conversely, in mixed, axonal-demyelinating CTS cases indicating low-grade nerve conduction blockade, it is reasonable to conduct kinesiotherapy [2], together with neuromobilization procedures of the median nerve [67,68,69], which can achieve functional improvement [22], supplemented with corticosteroid injections [70]. Kinesiotherapy and neuromobilizations are recommended to increase mobility in the wrist and improve the axoplasmic flow within the intrinsic nerve neurotubules and neurofilaments, both anterogradely and retrogradely [68,71]. In cases of ineffectiveness in conservative CTS treatment, it is accepted that when lesions of an axonal-demyelination nature are detected in the motor median nerve fibers in ENG studies, surgical decompression should be applied [1,35,72].

## 5. Conclusions

Our results provide insight into the symptoms and abnormalities in neurophysiological tests most frequently detected in a random, general population of patients with CTS, without their initial selection burdened with the subjective nature of clinical assessment. Our study demonstrates that, in this population of CTS patients without chronic comorbidities—diagnosed based on ENG results qualifying the disease as extreme or severe according to Padua et al.’s classification [44,46], where the absence of SNAP recording is a characteristic feature—the outcomes of the Phalen and Tinel tests are not equivocal. Our results demonstrate the superior utility of Phalen’s test in the clinical diagnosis of mild CTS, with positive results that are moderately correlated with amplitude abnormalities rather than distal latency parameters in SCV studies. Positive Tinel’s test results are more useful for diagnosing CTS patients with advanced axonal and demyelinating changes in the motor fibers of the median nerve. The correlation between clinical and functional neurophysiological test results allows us to not only determine the source and severity of median nerve fiber pathology but also to personalize the process of conservative and subsequent surgical treatment—and vice versa—depending on CTS severity. Neurophysiological studies are highly reliable, and practitioners who wish to rely solely on physical examination risk depending on an inherently less objective assessment.

## Figures and Tables

**Table 1:** Demographic and anthropometric characteristics of the subjects enrolled in the study. Minimum and maximum ranges, mean values, and standard deviations (SDs) are presented.

Study Group Variable	Healthy Volunteers (Control) N = 75 ♀ = 53, ♂ = 22		CTS Patients N = 75 ♀ = 52, ♂ = 23		Control vs. Patients
	Mean ± SD	Min–Max	Mean ± SD	Min–Max	*p*-Value
Age (years)	57.6 ± 12.9	18–60	56.4 ± 9.6	18–59	0.07
Height (cm)	166.4 ± 8.2	156–181	165.7 ± 7.1	155–182	0.08
Weight (kg)	56.3 ± 8.4	48.5–87.0	57.3 ± 8.1	46.3–85.1	0.09
BMI	20.3		20.9		0.08

Abbreviations: CTS—carpal tunnel syndrome.

**Table 2:** Comparison of results from median nerve electroneurographic studies in groups of healthy subjects (N = 75) and CTS patients (N = 75). Ranges and mean values with standard deviations are presented. *p*—significant differences are marked in bold.

Test Conditions	Parameter	Controls	CTS Patients	*p*
		Non-Injured Upper Extremities CMAP n = 112 SNAP n = 112	Symptomatic Side CMAP n = 108 SNAP n = 66	
CMAP (M-wave) Stimulation at wrist, APB recording	Amplitude (µV)	5000–12050 7325.4 ± 944.2	400–13500 5440.2 ± 530.2	**0.04**
	Latency (ms)	2.8–4.1 3.6 ± 0.2	2.7–10.7 5.5 ± 1.5	**0.03**
SNAP (SCV) Stimulation of 2nd finger, recording at wrist	Amplitude (µV)	10.4–28.6 18.8 ± 1.3	8.9–15.9 5.9 ± 1.3	**0.009**
	Latency (ms)	2.7–3.8 3.1 ± 0.2	2.5–5.4 4.1 ± 0.7	**0.04**

Abbreviations: CMAP (M-wave)—compound muscle action potential recording; APB—abductor pollicis brevis muscle; ENG—electroneuronography; SNAP (SCV)—sensory nerve action potential in sensory conduction studies; CTS—carpal tunnel syndrome.

**Table 3:** Distribution of Phalen and Tinel test results according to SNAP latency ranges (1 ms intervals) in patients with CTS (n = 112 symptomatic hands). Results are shown separately for patients with recordable SNAPs (n = 66) and those without measurable responses (n = 46; NA). Lack of SNAP recordings reflects advanced stages of CTS, when sensory potentials are not frequently recorded.

All Results n = 112					
Test	Patients with Recorded SNAP (n = 66)				Patients Without Recorded SNAP
	n = 7	n = 26	n = 27	n = 6	n = 46
	SNAP latency (ms)				
	≤3	≤4	≤5	≤6	NA
Phalen positive	0	10	15	3	27
Phalen negative	7	16	12	3	19
Tinel positive	0	7	7	2	23
Tinel negative	7	19	20	4	23

Abbreviations: SNAP (SCV)—sensory nerve action potential in sensory conduction studies.

**Table 4:** Cross-tabulation of Phalen and Tinel test results in 112 symptomatic hands examined for CTS detection. Values represent the number of hands with positive and negative outcomes in both tests.

Test	Tinel Positive	Tinel Negative	All Tests
**Phalen positive**	27	28	55
**Phalen negative**	12	45	57
**All tests**	39	73	112

## Data Availability

All the data generated or analyzed during this study are included in the published article.
